# Predictors of mortality for patients with COVID-19 and large vessel occlusion

**DOI:** 10.1177/1591019920954603

**Published:** 2020-08-30

**Authors:** David J Altschul, Charles Esenwa, Neil Haranhalli, Santiago R Unda, Rafael de La Garza Ramos, Joseph Dardick, Jenelys Fernandez-Torres, Aureliana Toma, Daniel Labovitz, Natalie Cheng, Seon-Kyu Lee, Allan Brook, Richard Zampolin

**Affiliations:** 1 Department of Neurosurgery Montefiore Medical Center, Albert Einstein College of Medicine, New York, NY, USA; 2 Department of Neurology Montefiore Medical Center, Albert Einstein College of Medicine, New York, NY, USA; 3 Albert Einstein College of Medicine, New York, NY, USA; 4 Department of Radiology Montefiore Medical Center, Albert Einstein College of Medicine, New York, NY, USA

**Keywords:** Ischemic stroke, COVID-19, emergent large vessel occlusion, outcomes, mechanical thrombectomy

## Abstract

**Background:**

This study evaluates the mortality risk of patients with emergent large vessel occlusion (ELVO) and COVID-19 during the pandemic.

**Methods:**

We performed a retrospective cohort study of two cohorts of consecutive patients with ELVO admitted to a quaternary hospital from March 1 to April 17, 2020. We abstracted data from electronic health records on baseline, biomarker profiles, key time points, quality measures and radiographic data.

**Results:**

Of 179 patients admitted with ischemic stroke, 36 had ELVO. Patients with COVID-19 and ELVO had a higher risk of mortality during the pandemic versus patients without COVID-19 (OR 16.63, p = 0.004). An age-based sub-analysis showed in-hospital mortality in 60% of COVID-19 positive patients between 61-70 years-old, 66.7% in between 51-60 years-old, 50% in between 41-50 years-old and 33.3% in between 31-40 years old. Patients that presented with pulmonary symptoms at time of stroke presentation had 71.4% mortality rate. 27.3% of COVID-19 patients presenting with ELVO had a good outcome at discharge (mRS 0-2). Patients with a history of cigarette smoking (p = 0.003), elevated d-dimer (p = 0.007), failure to recanalize (p = 0.007), and elevated ferritin levels (p = 0.006) had an increased risk of mortality.

**Conclusion:**

Patients with COVID-19 and ELVO had a significantly higher risk for mortality compared to COVID-19 negative patients with ELVO. A small percentage of COVID-19 ELVO patients had good outcomes. Age greater than 60 and pulmonary symptoms at presentation have higher risk for mortality. Other risk factors for mortality were a history of cigarette smoking, elevated, failure to recanalize, elevated d-dimer and ferritin levels.

## Introduction

Although the novel coronavirus (COVID-19) is predominantly a disease affecting the lower respiratory tract, recent studies have also reported neurological findings associated with this disease.^1,2^ Two studies showed that cerebrovascular accidents (mainly ischemic stroke) were more common among severe COVID-19 patients.^2,3^ A recent report also from New York City described five cases of COVID-19 patients presenting with large vessel occlusion.^4^ Practicing stroke specialists are familiar with incorporating pre-functional status into clinical decision-making paradigms when considering emergent treatments such as intravenous (IV) tissue-plasminogen activator (tPA) or mechanical thrombectomy (MT).^5^ It is unclear how COVID-19 status effects mortality in patients with emergent large vessel occlusion (ELVO), and how this should guide clinical treatment decisions.

The aim of this study is to evaluate the impact of COVID-19 on patients with ELVO, as it relates to outcomes and mortality compared to patients without COVID-19 during the same epoch of time during the pandemic. Our hypothesis is that ELVO patients with COVID-19 have a higher rate of mortality compared to ELVO patients without COVID.

## Methods

### Study design and participants

This study was approved by our institutional review board. Patient informed consent was waived. The data that support the findings of the study are available from the corresponding author upon reasonable request. This retrospective cohort study included two cohorts of patients admitted to one of three major hospitals of our healthcare network including, an academic medical center and comprehensive stroke center, which accepts transfers for complex cases from eight community hospitals, during March 1 to April 17, 2020. All patients diagnosed with ELVO were included. ELVO was defined as acute focal neurological deficits with an associated occlusion of the internal carotid artery, M1 segment middle cerebral artery, M2 segment middle cerebral artery, or basilar artery with radiologic confirmation by computed tomography angiography (CTA). We also included patients with occlusions of anterior cerebral artery, posterior cerebral artery or vertebral artery and labelled them as “other” large vessel occlusion.

### Case ascertainment and study variables

Cases were ascertained by primary and secondary ICD10 code for ischemic stroke and ELVO cases were identified through neuroradiology logs. Data abstracted from medical records included demographics, clinical variables, laboratory values, neuroimaging findings (initial Alberta Stroke Program Early CT Score [ASPECTS], location of ELVO, stroke treatment time points and results as they pertain to MT, outcome variables (hemorrhagic transformation, good functional (mRS 0–2), poor functional (mRS 3–5) outcome at discharge, and in-hospital mortality). Pulmonary symptoms at presentation were recorded and defined as fever, hypoxia, or prototypical abnormal CT chest findings.

### COVID-19 screening and diagnosis

Cases were defined as positive by detection of viral RNA using real-time reverse transcriptase–polymerase chain reaction (RT-PCR) assay testing, performed within the hospital system or documented at an outside system prior to transfer. All patients admitted with a stroke diagnosis during the inclusion period were screened for COVID-19.

### Statistical analysis

Baseline characteristics between groups, were compared performing parametric and non-parametric analyses with the *t* test, *χ*^2^ test, and Mann-Whitney *U* test as appropriate. No imputation was made for missing data. Unfavorable outcomes with *χ*^2^ test p < 0.05 were analyzed through univariate and multivariate logistic regression controlling for explanatory variables with p < 0.25 on univariate analysis was done if unadjusted model had a p < 0.05. Laboratory values were not considered for multivariate models due to missing data. All data analyses were conducted in IBM SPSS (vs26.0, Armonk, NY: IBM Corp).

## Results

We found a total of 36 patients with ELVO during the entire study period, 36% (n = 13) tested positive for COVID-19 (Table 1). Patients with COVID-19 and ELVO had higher rates of in-hospital mortality (63% vs 9%, p = 0.001) (Table 1). They also had significantly higher mortality risk compared to patients without COVID-19 during the pandemic (OR: 16.63 95% CI: 2.47–111.79, p = 0.004) (Table 1), still significant when adjusted for age and comorbidities (OR: 15.13 95% CI: 2.08–110.05, p = 0.007).

**Table 1.** COVID-19 positive versus negative for COVID-19 from March to April 2020.

Variables	COVID-19 negative (n = 23)	COVID-19 positive (n = 13)	P-value
Age-yr., mean (SD)	68.3 (13.69)	63 (11.87)	0.247#
Male sex, n (%)	13 (56.5)	5 (38.5)	0.298
Race			
White, n (%)	7 (30.4)	1 (7.7)	0.445
African American, n (%)	7 (30.4)	5 (38.5)	
Hispanic, n (%)	7 (30.4)	6 (46.2)	
Other, n (%)	2 (8.7)	1 (7.7)	
Vascular risk factors			
Hypertension, n (%)	18 (78.3)	9 (69.2)	0.548
Hyperlipidemia, n (%)	12 (52.2)	5 (38.5)	0.429
Diabetes, n (%)	8 (34.8)	6 (46.2)	0.501
Atrial fibrillation, n (%)	4 (17.4)	2 (15.4)	0.877
Congestive heart failure, n (%)	3 (13)	0 (0)	0.174#
Smoking, n (%)	3 (13)	3 (23.1)	0.646
Clinical presentation†			
mRS baseline, median (IQR)	0 (0–2)	0 (0–1)	0.494
NIHSS on admission, median (IQR)	16 (12–20)	16 (14–20)	0.871
ASPECTS on admission, median (IQR)	7 (4–8)	6 (3–8)	0.960
Laboratory values on admission‡			
White blood cell-count, median (IQR) per mm^3^	8.4 (6.4–11.3)	7.4 (5.2–9.4)	0.379
Platelet-count, median (IQR) k per mm^3^	212 (188–249)	249 (178–306)	0.239
Prothrombin time, median (IQR) sec	13.9 (13.2–15.1)	14.3 (13.6–15.6)	0.345
D-dimer, median (IQR) ng/ml	1.51 (.50–8.07)	20 (3.5–20)	0.055
Fibrinogen, median (IQR)	449 (388–578.5)	384 (198–561)	0.610
Ferritin, median (IQR)	593.5 (339–679)	1150 (640–2309)	0.260
Blood urea nitrogen, median (IQR)	15 (11–18)	12 (10–24)	0.770
Creatinine, median (IQR)	1.00 (.80–1.2)	.70 (.60–.90)	0.013
Location of occlusion			
Right MCA, n (%)	6 (26.1)	4 (30.8)	0.947
Left MCA, n (%)	10 (43.5)	5 (38.5)	0.695
Right ICA (with carotid T), n (%)	1 (4.3)	1 (7.7)	0.626
Left ICA (with carotid T), n (%)	1 (4.3)	1 (7.7)	0.626
Right ICA, n (%)	2 (8.7)	2 (15.4)	0.355
Left ICA, n (%)	4 (17.4)	1 (7.7)	0.657
Basilar, n (%)	1 (4.3)	0 (0)	0.366
Other LVO (ACA, PCA, Vertebral)	3 (13)	2 (15.4)	0.606
Treatment information¶			
Thrombolysis IV, n (%)	3 (13)	5 (38.5)	0.078
Thrombectomy, n (%)	9 (39.1)	3 (23.1)	0.326
*Time points*			
Emergency patients			
Onset to first hospital, mins, median (IQR)	342 (149–988)	870 (161–1560)	0.621
Onset to our institution, mins, median (IQR)	604 (303–1160)	579 (124–1707)	0.655
>6hs from onset to door, n (%)	15 (65.2)	3 (50)	0.494
Thrombectomy patients			
Door to groin, mins, median (IQR)	84 (68–110)	99 (69–126)	0.797
Groin to recanalization, mins, median (IQR)	38 (33–56)	60 (31–68)	0.727
TICI score ≥2b, n (%)	8 (88.9)	2 (66.7)	0.371
Day 1 NIHSS, median (IQR)	13 (8–21)	4 (1–18)	0.060
Outcomes#			
Hemorrhagic transformation, n (%)	1 (4.3)	2 (20)	0.151
Good outcomes (mRS 0-2), n (%)	4 (19)	3 (27.3)	0.001
Poor outcomes (mRS 3-5), n (%)	15 (71.4)	1 (9.1)	
In-hospital mortality, n (%)	2 (9.5)	7 (63.6)	
Logistic regression	Poor outcomes at discharge (mRS 3-5)	In-hospital mortality	
COVID-19 negative *(reference)*	Unadjusted OR [95% CI] P value		
COVID-19 positive	.089 [.007–1.102] p = 0.060	16.63 [2.47-111.79] p = 0.004	
Multivariate Logistic regression	In-hospital mortality		
COVID-19 negative *(reference)*	Adjusted OR [95% CI] P value		
COVID-19 positive	15.13 [2.08–110.05] p = 0.007		

^*^Included in multivariate logistic regression.

^†^Excluded for posterior circulation: ASPECTS 1/36 (2.8%).

^‡^Missing data: D-dimer 18/36 (50%), Fibrinogen 26/36 (72.2%), Ferritin 23/36 (68.9%), Prothrombin time 2/36 (5.6%).

^#^Missing data: discharge mRS 4/36 (11.1%).

^¶^Missing data: Onset to first hospital 5/30 (16.7%), Onset to our institution 1/30 (3.3%).

**Figure 1.**
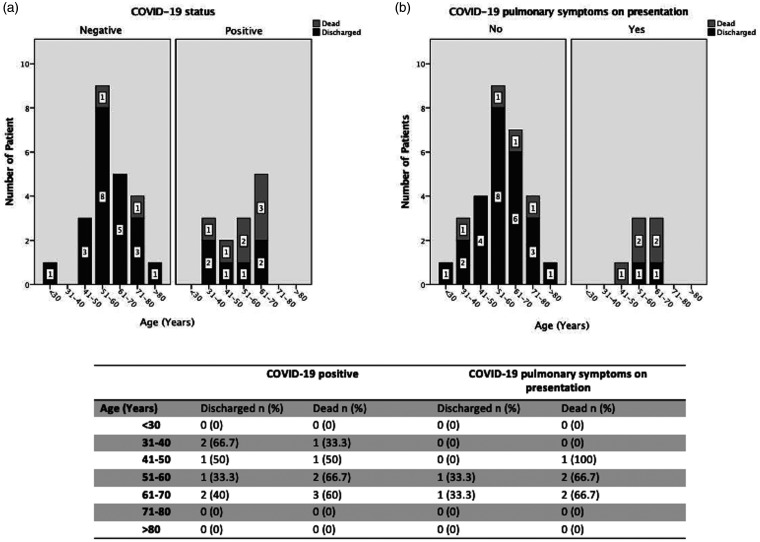
(a) Age breakdown for in-hospital mortality in patients tested positive for COVID-19 and (b) in patients with COVID-19 pulmonary symptoms on presentation.

The age breakdown sub-analysis showed in-hospital mortality in 60% of COVID-19 positive patients between 61–70 years-old, 66.7% in between 51–60 years-old, 50% in between 41–50 years-old and 33.3% in between 31–40 years old (Figure 1(a)). 53.8% of the COVID-19 patients presented with pulmonary symptoms (Figure 1(b)). Within this group 71.4% of these patients expired (Figure 1(b).

**Table 2.** Discharge and deaths breakdown by age in patients tested positive for COVID-19 and patients with pulmonary symptoms on presentations (Supplement to Figure 1(a) and (b)).

	COVID-19 positive		COVID-19 pulmonary symptoms on presentation	
Age (years)	Discharged n (%)	Dead n (%)	Discharged n (%)	Dead n (%)
<30	0 (0)	0 (0)	0 (0)	0 (0)
31–40	2 (66.7)	1 (33.3)	0 (0)	0 (0)
41–50	1 (50)	1 (50)	0 (0)	1 (100)
51–60	1 (33.3)	2 (66.7)	1 (33.3)	2 (66.7)
61–70	2 (40)	3 (60)	1 (33.3)	2 (66.7)
71–80	0 (0)	0 (0)	0 (0)	0 (0)
>80	0 (0)	0 (0)	0 (0)	0 (0)

On univariate analysis for predictors for mortality, we also found that elevated d-dimer (median 20 ng/ml vs. 1.51 ng/ml, p = 0.007), a history of cigarette smoking (n = 5, 55.6% vs. n = 1, 3.7%, p = 0.003), increased ferritin (median 2426 ng/ml vs. 628 ng/ml, p = 0.006), failure to recanalize (TICI >2 b n = 1, 33.3% vs. n = 9, 100%) were all significantly associated with mortality (Table 2). On multivariate analysis pulmonary symptoms (OR 20.81 [3.26–133.03]), p = 0.001), and d-dimer (OR 1.21 [1.04–1.39], p = 0.012) were significant predictors of mortality (Table 3).

**Table 2.** Univariate analysis of predictors of in-hospital mortality for patients with ELVO.

Variables	Discharged (n = 27)	Dead (n = 9)	P value
Age-yr., mean (SD)	65.89 (13.52)	68 (12.6)	0.683
Male sex, n (%)	14 (51.9)	4 (44.4)	0.700
Pulmonary symptoms, n (%)	3 (11.1)	6 (66.7)	0.001
Race			
White, n (%)	6 (22.2)	2 (22.2)	0.118
African American, n (%)	11 (40.7)	1 (11.1)	
Hispanic, n (%)	7 (25.9)	6 (66.7)	
Other, n (%)	3 (11.1)	0 (0)	
Vascular risk factors			
Hypertension, n (%)	23 (85.2)	4 (44.4)	0.015
Hyperlipidemia, n (%)	13 (48.1)	4 (44.4)	0.847
Diabetes, n (%)	9 (33.3)	5 (55.6)	0.236
Atrial fibrillation, n (%)	5 (18.5)	1 (11.1)	0.606
Congestive heart failure, n (%)	3 (11.1)	0 (0)	0.296
Smoking, n (%)	1 (3.7)	5 (55.6)	0.003
Clinical presentation			
mRS baseline, median (IQR)	0 (0–1)	0 (0–2)	0.747
NIHSS on admission, median (IQR)	15 (10–20)	19 (17–20)	0.117
ASPECTS on admission, median (IQR)	7 (4–8)	6 (2–8)	0.446
Laboratory values on admission			
White blood cell-count, median (IQR) per mm^3^	8.4 (5.7–10.7)	8.7 (5.2–10.5)	0.943
Platelet-count, median (IQR) k per mm^3^	217 (188–256)	220 (178–250)	0.858
Prothrombin time, median (IQR) sec	13.9 (13.5–15.1)	13.8 (13.5–15.6)	0.827
D-dimer, median (IQR) ng/ml	1.51 (.50–8.86)	20 (19.3–20)	0.007
Fibrinogen, median (IQR)	420 (362–485)	379.5 (137–696.5)	0.914
Ferritin, median (IQR)	628 (119–691)	2426 (1729–3075)	0.006
Blood urea nitrogen, median (IQR)	14 (10–18)	23 (11–26)	0.312
Creatinine, median (IQR)	1.00 (.70–1.2)	.80 (.60–.90)	0.032
Location of occlusion			
Right MCA, n (%)	8 (29.6)	2 (22.2)	0.667
Left MCA, n (%)	11 (40.7)	4 (44.4)	0.845
Right ICA (with carotid T), n (%)	2 (7.4)	0 (0)	0.401
Left ICA (with carotid T), n (%)	0 (0)	2 (22.2)	0.012
Right ICA, n (%)	3 (11.1)	1 (11.1)	1.0
Left ICA, n (%)	3 (11.1)	2 (22.2)	0.404
Basilar, n (%)	1 (3.7)	0 (0)	0.558
Other LVO (ACA, PCA, Vertebral)	3 (11.1)	2 (22.2)	0.151
Treatment information			
Thrombolysis i.v, n (%)	6 (22.2)	2 (22.2)	1.0
Thrombectomy, n (%)	9 (33.3)	3 (33.3)	1.0
Time points			
Emergency patients			
Onset to first hospital, mins, median (IQR)	342.5 (149–988)	870 (161–1312)	0.767
Onset to our institution, mins, median (IQR)	582 (245–1136)	998 (235–1577)	0.716
>6hs from onset to door, n (%)	15 (62.5)	3 (60)	0.917
Thrombectomy patients			
Door to groin, mins, median (IQR)	84 (68–99)	126 (69–134)	0.373
Groin to recanalization, mins, median (IQR)	38 (31–56)	60 (36–68)	0.482
TICI score ≥2b, n (%)	9 (100)	1 (33.3)	0.007
Day 1 NIHSS, median (IQR)	11 (6–12)	19 (1–23)	0.482

**Table 3.** Multivariate analysis for predictors of in-hospital mortality.

Multivariate analysis	OR [95% CI] P values
Pulmonary symptoms	20.81 [3.26–133.03] p = 0.001
Hypertension	.471 [.11–2.05] p = 0.316
Current smoker	1.71 [.27–10.97] p = 0.570
Elevated d-dimer	1.21 [1.04–1.39] p = 0.012
Elevated Ferritin	1.004 [.99–1.01] p = 0.143
Elevated Creatinine	.622 [.027–14.39] p = 0.767

## Discussion

We aimed to evaluate the effect of the COVID-19 on ELVO patients on patient outcomes with focus on mortality risk. We evaluated sociodemographic data, comorbidities, established treatment time parameters, established imaging parameters, neurologic outcomes, and mortality between the two groups.

The mortality for patients with ELVO and COVID-19 was extremely high (63.6%), however a minority did survive with good outcome (27.3%) (Table 1). Poor outcomes were significantly less in the COVID-19 positive group however it appears this is likely secondary to the fact that these patients who would otherwise survive with poor outcomes expired secondary to COVID-19. COVID-19 patients that presented with pulmonary symptoms had the highest risk of death (71.4%) (Figure 1). The mortality rate in this series was quite similar to a group in Paris.^6^ They concluded that COVID-19 was not primarily responsible for stroke during the pandemic. They also reported that 70% of stroke patients had mild or no respiratory symptoms. D-dimer levels were much higher in the COVID-19 positive group (20 ng/ml) versus the COVID-19 negative group (1.51 ng/ml), which did not reach clinical significance however given the small sample size is likely the reason as other studies have shown this value to be elevated in stroke patients with COVID-19. Elevated d-dimer along with ferritin was significantly associated with increase mortality on univariate analysis however only d-dimer remained significant on multivariate analysis. A history of cigarette smoking was also a predictor of mortality on univariate analysis however was not significant on multivariate analysis. This may be due to the small sample size as these findings are similar to previously reports in the literature.^7^ The presence or absence of gastrointestinal symptoms was not addressed in this cohort and is still an area of future research.

It is unclear how to incorporate COVID-19 status into the clinical decision making for evaluation of ELVO patients. Careful consideration is warranted in deciding to move forward with thrombectomy in patients with ELVO that also are presenting with pulmonary findings of COVID-19, and patients beyond the age of 60 as these patients have particularly high mortality rates (Figure 1). COVID-19 status alone should not preclude a patient from thrombectomy or thrombolysis, as we had ELVO patients survive from ages 30–70 with COVID-19, however careful counselling with family regarding the prognosis is warranted. The limitations of this study are its retrospective and single-center design, lack of long-term outcome follow up, and the sample size is small. Future clinical outcomes research is needed with larger sample sizes to help guide treatment paradigms.
